# Neuroinflammation and related neuropathologies in APP_SL_ mice: further value of this *in vivo* model of Alzheimer’s disease

**DOI:** 10.1186/1742-2094-11-84

**Published:** 2014-05-01

**Authors:** Tina Löffler, Stefanie Flunkert, Daniel Havas, Cornelia Schweinzer, Marni Uger, Manfred Windisch, Ernst Steyrer, Birgit Hutter-Paier

**Affiliations:** 1 QPS-Austria GmbH, Parkring 12, 8074 Grambach, Austria; 2 Institute of Molecular Biology and Biochemistry, Medical University Graz, Harrachgasse 21, 8010 Graz, Austria; 3 Amorfix Life Sciences Ltd, 3403 American Drive, Ontario, Canada L4V 1 T4

**Keywords:** Aβ peptides, Aβ oligomers, Microgliosis, Astrocytosis, Lipid peroxidation, Correlation analysis, Transgenic mice, APP_SL_

## Abstract

**Background:**

Beyond cognitive decline, Alzheimer’s disease (AD) is characterized by numerous neuropathological changes in the brain. Although animal models generally do not fully reflect the broad spectrum of disease-specific alterations, the APP_SL_ mouse model is well known to display early plaque formation and to exhibit spatial learning and memory deficits. However, important neuropathological features, such as neuroinflammation and lipid peroxidation, and their progression over age, have not yet been described in this AD mouse model.

**Methods:**

Hippocampal and neocortical tissues of APP_SL_ mice at different ages were evaluated. One hemisphere from each mouse was examined for micro- and astrogliosis as well as concomitant plaque load. The other hemisphere was evaluated for lipid peroxidation (quantified by a thiobarbituric acid reactive substances (TBARS) assay), changes in Aβ abundance (Aβ38, Aβ40 and Aβ42 analyses), as well as determination of aggregated Aβ content (Amorfix A^4^ assay). Finally, correlation analyses were performed to illustrate the time-dependent correlation between neuroinflammation and Aβ load (soluble, insoluble, fibrils), or lipid peroxidation, respectively.

**Results:**

As is consistent with previous findings, neuroinflammation starts early and shows strong progression over age in the APP_SL_ mouse model. An analyses of concomitant Aβ load and plaque deposition revealed a similar progression, and high correlations between neuroinflammation markers and soluble or insoluble Aβ or fibrillar amyloid plaque loads were observed. Lipid peroxidation, as measured by TBARS levels, correlates well with neuroinflammation in the neocortex but not the hippocampus. The hippocampal lipid peroxidation correlated strongly with the increase of LOC positive fiber load, whereas neocortical TBARS levels were unrelated to amyloidosis.

**Conclusions:**

These data illustrate for the first time the progression of major AD related neuropathological features other than plaque load in the APP_SL_ mouse model. Specifically, we demonstrate that microgliosis and astrocytosis are prominent aspects of this AD mouse model. The strong correlation of neuroinflammation with amyloid burden and lipid peroxidation underlines the importance of these pathological factors for the development of AD. The new finding of a different relation of lipid peroxidation in the hippocampus and neocortical regions show that the model might contribute to the understanding of complex pathological mechanisms and their interplay in AD.

## Background

Alzheimer’s disease (AD) is characterized by various neuropathological events including amyloid plaques, oxidative stress, and neuroinflammation. Dysregulated amyloid precursor protein (APP) metabolism and resulting Aβ generation seem to be central and early events in the disease [1]. Enhanced production of Aβ40 and Aβ42 trigger the onset of several pathological changes, in particular neuroinflammation. Activated microglia and reactive astrocytes represent the pivotal pathological hallmarks of neuroinflammation. Microgliosis is well known to be activated by Aβ, Aβ fibrils, and Aβ oligomers and occurs early in AD [2-5]. Intriguingly, early microgliosis promotes Aβ clearance by protofibrillar Aβ phagocytosis [6,7], whereas chronic microgliosis seems to promote Aβ accumulation and subsequent neurodegeneration [6]. Astrogliosis, similar to microgliosis, can be caused by Aβ fibrils or oligomers [8,9]. Transplanted murine adult and neonatal astrocytes are able to internalize and degrade aggregated human Aβ in the hippocampi of APP transgenic mice, and can thus serve as active Aβ clearing cells [10]. In contrast, it has been shown that cytokines and Aβ42 promote endogenous Aβ production in astrocytes [11]. Since the quantity of astrocytes exceeds that of neurons in the brain, activated astrocytes may consequently represent a significant source of Aβ during the neuroinflammatory processes [11]. Taken together, the current data indicate that reactive microglia and activated astrocytes possess both neuroprotective as well as neurodegenerative properties, the latter especially in the chronic manifestation of AD. Neuroinflammation promotes not only further Aβ expression but also oxidative stress. However, it is still under debate whether oxidative stress is a consequence of neurodegeneration [12]. Moreover, co-occurrence of neuroinflammation and oxidative stress markers leads to enhanced Aβ generation [13], thus closing the circuit between reciprocal interferences of neuropathological changes in AD.

Dysregulated APP metabolism can be triggered by mutations in different genes [14], such as the Swedish (K670N/M671L) [15] and London mutations (V717I) within the APP gene [16]. In humans the Swedish mutation causes a classic AD-like neuropathology, including amyloid plaque load, neurite dystrophy, neuronal loss, neuroinflammation, oxidative stress [17], and cognitive decline [18]. The London mutation, on the other hand, modifies the γ-secretase cleavage site and therefore shifts amyloid secretion towards a relative increase of Aβ42 [19], resulting in early memory deficits and impaired shifting abilities in humans [20]. The combination of both mutations leads not only to increased Aβ plaque pathology, but also to a strong and progressive decline of cognitive and social abilities in mice [21,22]. So far, several distinct neuropathological features of APP_SL_ mice that neuronally express human APP with both the Swedish and London mutations have not yet been described, although this murine model has been extensively used in neuropharmacology during the last decade ([23], review). Accordingly, we analyzed levels of soluble and insoluble Aβ species, aggregated Aβ, fibrillar Aβ, and oxidative stress markers. As a main goal, concomitant hallmarks for astrocytosis (GFAP) and microgliosis (CD11b), both indicators of neuroinflammation, were quantified in the hippocampus and neocortex of APP_SL_ mice. Correlational analyses comparing neuroinflammation markers with Aβ levels as well as fibrillar Aβ and levels of lipid peroxidation during disease progression were performed in this murine AD model.

## Methods

### Animals

APP_SL_ transgenic mice express APP751 with Swedish (K670N/M671L) and London (V717I) mutations. These mice feature characteristics of other already published APP Swedish and London mouse models [21,24], but are bred heterozygously on a pure C57BL/6 background at QPS-Austria. Non-transgenic littermates (ntg) served as the control for all experiments. A balanced number of male and female animals were used and housed in individually ventilated cages under a 12 hour light and dark cycle. Standard rodent chow (Altromin™ Lage, Germany) and normal tap water were available to the animals *ad libitum*. Room temperature and humidity were kept at approximately 24°C and between 40 and 70%, respectively. Mice of different ages (between 1 and 12 months) were selected for measurements; actual numbers are given in the figure legends. Animal studies conformed to the Austrian guidelines for the care and use of laboratory animals and were approved by the Styrian Government, Austria (FA10A-78Jo-71-2010).

### Tissue sampling

Animals were deeply sedated by standard inhalation anesthesia and transcardially perfused with 0.9% NaCl. Brains were carefully removed and hemisected. The left hemispheres were further dissected into the neocortex and hippocampus, immediately shock frozen on dry ice and stored at -80°C for biochemistry. The right hemispheres were immersion fixed in 4% paraformaldehyde/PBS (pH 7.4) at room temperature for 1 hour, cryo-conserved in 15% sucrose solution until sunk and shock frozen in liquid isopentane. Tissues were stored at -80°C until used for histological analyses.

### Tissue homogenization and protein extraction

Frozen tissue samples were weighed and a tissue homogenization buffer (THB) (250 mM Sucrose, 1 mM EDTA, 1 mM EGTA, 20 mM Tris pH 7.4) including 1 × protease inhibitor (Calbiochem, Darmstadt, Germany) was added, 1 ml THB per 100 mg of cortical tissue and 2.5 ml per 100 mg of hippocampal tissue. The tissue was homogenized with a tissue ruptor (Qiagen, Hilden, Germany) for 20 seconds at 33,000 rpm.

For the extraction of soluble Aβ peptides (‘aqueous-soluble’ fraction), 100 μl of the THB homogenate was mixed with 100 μl diethylamine (DEA) solution (0.4% DEA, 100 mM NaCl). The mixture was spun for 1 hour at 74,200 × g [m/s^2^] at 4°C. 170 μl of the supernatant was transferred to a 1.5 ml Eppendorf tube and neutralized with 17 μl 0.5 M Tris, at a pH 6.8.

For the extraction of insoluble Aβ peptides (’formic acid soluble’ fraction), 100 μl of the THB homogenate was mixed with 220 μl cold formic acid (FA) and sonicated for 1 minute on ice. Following this, 300 μl of the solution was transferred to a tube and spun for 1 hour at 74,200 × g [m/s^2^] at 4°C. Thereafter, 210 μl of the supernatant was placed into a fresh tube, using a pipette, and mixed with 4 ml formic acid (FA) neutralization solution (1 M Tris, 0.5 M Na_2_HPO_4_, 0.05% NaN_3_).

### Measurement of Aβ concentrations

Samples were analyzed for Aβ38, Aβ40 and Aβ42 with the MSD™ 96-well MULTI-SPOT™ 4G8 Abeta Triplex Assay (Meso Scale Discovery, Rockville, Maryland, United States) detecting human and rodent Aβ species. The assay was performed according to the manual. Plates were analyzed on the Sector Imager 2400 (MesoScale Diagnostics™, Rockville, Maryland, United States).

### Measurement of lipid peroxidation -TBARS assay

In order to measure the lipid peroxidation, 50 μl of the THB homogenates and 50 μl of 5% sodium dodecyl sulfate (SDS) solution, including 1 × protease inhibitor as well as 5 mM of 1 × butylhydroxytoluene (BHT) solution, were mixed and sonicated for 5 seconds. Malondialdehyde (MDA) served as the standard at final concentrations between 1 and 20 μM in 1:1 (v/v) mixtures of THB and 5% SDS. The thiobarbituric acid reactive species assay (TBARS-assay) was started by adding 55 μl of 1.33% TBA and 95 μl of 20% acetic acid (pH 3.5) to 100 μl of the prepared samples or standards. After 1 hour of incubation at 95°C, 250 μl of n-butanol and pyridine mixture (15:1 v/v) was added and gently mixed by inverting the tubes. After centrifugation for 10 minutes at 4000 × g [m/s^2^], 200 μl of the upper organic phase was transferred to 96-well plates and absorbance was measured at 535 nm with a μQuant plate photometer (BioTek Instruments Inc., Winooski, Vermont, United States). It has to be mentioned that the TBARS assay not only detects MDA, but reacts with other species, including non-lipid derived MDA or lipid-derived non-MDA substances. To differentiate these species, other supportive analyses would be needed. However, these types of analyses would go beyond the intention of this study to show the most common read-out for lipid peroxidation and necessitate a different basic setup.

### Aggregated Aβ quantification

Hippocampal and cortical homogenates were analyzed with the Amorfix Aggregated Aβ Assay (A^4^). In short, samples were diluted in an assay buffer (phosphate buffer, 1:100 for hippocampal samples, 1:1000 for neocortex samples) and enriched on the A^4^ matrix to allow the separation of Aβ monomers (flow-through) and Aβ aggregates (matrix-bound). After the enrichment process, samples were eluted from the A^4^ matrix and aggregated Aβ was dissociated into monomers. After incubation with a human-specific N-terminal Aβ antibody coupled to Europium™ beads (Amorfix, Mississauga, Ontario, Canada) followed by incubation with C-terminal Aβ antibodies (recognizing Aβ40 and 42) coupled to magnetic beads, time-resolved fluorescence was used to determine levels of human-aggregated Aβ species in each sample.

### Immunohistochemistry

Frozen brain hemispheres were sagittally cut. A systematic uniform random set of five sagittally cut 10-μm-thick mounted sections from five different medio-lateral levels (L2, L4, L6, L8 and L11, see Additional file 1 for sectioning protocol) per animal were labeled with the following primary antibodies: LOC recognizing amyloid fibrils and fibrillar oligomers, but not monomers (AB2287; 1:500 Millipore, Billerica, Massachusetts, United States), GFAP (Z0334; 1:500 Dako, Vienna, Austria) for reactive astrocytes and CD11b (MCA711;1:1000 AbDSerotec, Puchheim, Germany) for activated microglia. Binding was visualized using highly cross-absorbed Cy secondary antibodies (Cy2, 3 and 5; 1:500 Dianova, Hamburg, Germany). Whole slices were imaged on a Zeiss Z1 Axio Imager (Zeiss, Vienna, Austria) using an apochromat 10 × lens, narrow band filter sets fitting to the dyes, and an AxioCam MRm black and white camera (Zeiss, Vienna Austria) with 1 × opto-coupler. After delineation of the hippocampal and neocortical regions (Additional file 2 for region definition), stainings were quantified in automated rater independent procedures with Image-Pro™ Plus (MediaCybernetics, Rockville, Maryland, United States), taking into account the individual size of the area of interest in the slice.

Immunoreactive (IR) objects were detected above an intensity threshold based on 8-bit 256 grey levels, which was adaptive for LOC and GFAP IR (mean signal + 1.2 × standard deviation of mean signal) and constant for CD11b detection. The level varied for the different labelings adjusted to the background fluorescence in the channel. Furthermore, detected objects had to overcome a minimal size of 7 (LOC), 30 (GFAP - excludes single processes not related to a somata) or 150 μm^2^ (CD11b - excludes resting microglial labeling). The latter was based on frequency distribution statistics showing a shift towards activated over resting microglia at this size. Data export was as an automated filing mean and sum signal, as well as surface area percentage, object density, mean objects size, and threshold levels together with the image title. Region size did not differ among groups, excluding methodological bias and atrophy in the scope of the natural variance and used number (Additional file 3).

### Statistics

Data analysis and graphs were created in GraphPad Prism™ 4.03 (GraphPad Software Inc., California, United States). Graphs include group means and standard error of the mean (SEM). The significance level was set at *P* < 0.05. Group means were compared using a one-way analysis of variance (ANOVA) with a subsequent Newman-Keuls *post hoc* comparison test, or a two-way ANOVA and a Bonferroni’s multiple comparison test. Exact sample numbers are given in the figure legends. For histological measurements, the values of the five slices per animal were averaged to the individual mean. Group means were calculated by averaging the individual means.

## Results

### Aβ levels in APP_SL_ mice increase over age in cortical and hippocampal samples

**Figure 1:**
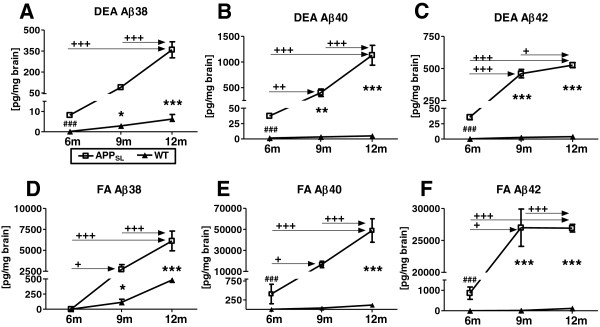
**Aβ concentrations in hippocampal brain homogenates of APP**_**SL **_**transgenic mice increase over age.** Aβ38 **(A, D)**; Aβ40 **(B, E)**, and Aβ42 **(C, F)** concentrations are shown in pg/mg hippocampal homogenates for soluble (DEA; **A**-**C**) and insoluble (FA; **D**-**F**) fractions of 6, 9, and 12-month-old APP_SL_ mice in comparison to non-transgenic littermates. n = 10/group. All data were analyzed by two way ANOVA followed by Bonferroni’s post hoc test. * significances between genotypes **+** significances between age groups of APP_SL_ transgenic mice. ^#^ significances between 6-month-old APP_SL_ and non-transgenic littermates as analyzed by Fisher’s least significance test. *P <0.05; **P <0.01; ***P <0.001. Abbreviations: amyloid beta: Aβ, diethyl amine: DEA, formic acid: FA, analysis of variance: ANOVA, Amyloid Precursor protein carrying the Swedish London mutation: APP_SL_, probability: P.

Aβ38, Aβ40 and Aβ42 levels in soluble (DEA) and insoluble (FA) fractions from the hippocampal tissue of 6, 9, and 12-month-old APP_SL_ animals were analyzed using a MesoScale Discovery™ 4G8 Abeta Triplex Assay. As expected, all Aβ levels were very low in non-transgenic mice at 6 months and increased only moderately until 12 months of age (Figure 1A). In APP_SL_ transgenic animals, all but Aβ38 levels increased significantly in the soluble fraction between 6 and 9 months of age, with the highest increase in the Aβ42 species (Figure 1B, C). Insoluble Aβ38 and Aβ40 progressively increased with age, resulting in levels of approximately 6000 pg/mg for Aβ38 and 50,000 pg/mg for Aβ40 at 12 months of age (Figure 1D, E). Interestingly, insoluble Aβ42 levels reached a plateau at the age of 9 months, with levels staying high at approximately 26,500 pg/mg at both 9 and 12 months of age (Figure 1F). A parallel progression could be observed for the soluble peptides (Figure 1A-C), though Aβ38, Aβ40 and Aβ42 levels were 17-, 45-, and 53-fold lower, respectively, compared to the insoluble Aβ in the FA fraction (Figure 1D-F).

The analyses of cortical samples of APP_SL_ mice revealed a similar trend. However, soluble Aβ levels were about 2-3-fold higher in the hippocampus than in the neocortex. Insoluble Aβ levels on the other hand, were almost as high in the neocortex as in the hippocampus (Additional file 4).

Additional analyses of all Aβ data by Fisher’s least significance test accounting for data with very strong progression, revealed a highly significant increase of soluble Aβ38, Aβ40 and Aβ42 as well as insoluble Aβ40 and Aβ42 levels in the hippocampus and neocortex of APP_SL_ mice (Figure 1 and Additional file 4). This analysis demonstrates that six-month-old APP_SL_ mice already exhibit significantly increased Aβ levels compared to non-transgenic littermates.

### Assessment of aggregated Aβ and fibrillar Aβ plaque load

**Figure 2:**
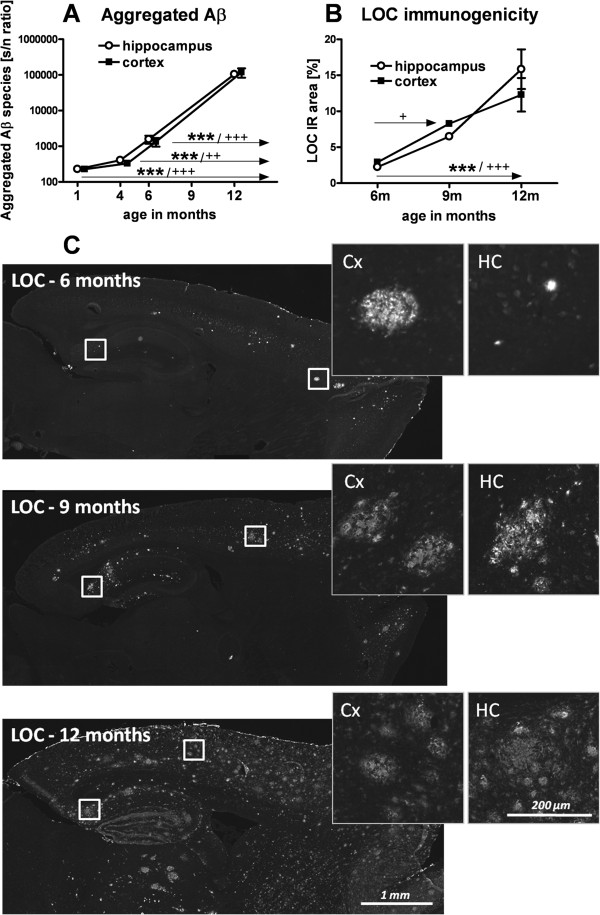
**Progressively increasing aggregated Aβ concentrations and plaque load in brain homogenates of APP**_**SL **_**transgenic mice. A**: Aggregated Aβ concentration in the hippocampus and neocortex of 1, 4, 6, and 12-month-old APP_SL_ transgenic mice as analyzed by Amorfix A^4^ assay. Data are shown as signal to noise (S/N) ratio, with N equal to the signal generated by buffer alone. Animals of the one month group were between 1 and 3-months-old. 1 month: n = 7; 4 months: n = 5; 6 and 12 months: n = 11. Data were analyzed by two way ANOVA followed by Bonferroni’s *post hoc* test. * significances between age groups of APP_SL_ neocortical tissue. **+** significances between age groups of APP_SL_ hippocampal tissue. **B**: Quantification of LOC as immunoreactive area in percent in the hippocampus and neocortex of 6, 9, and 12-month-old APP_SL_ transgenic mice. n = 6/group. **C**: Representative images of sagittal sections of 6, 9, and 12-month-old APP_SL_ transgenic mice labeled with LOC. Inlets show magnifications of neocortical and hippocampal brain regions. Data were analyzed by one way ANOVA followed by Bonferroni’s *post hoc* test. *P <0.05; **P <0.01; ***P <0.001. Abbreviations: amyloid beta: Aβ, analysis of variance: ANOVA, Amyloid Precursor protein carrying the Swedish London mutation: APP_SL_, probability: P, neocortex: Cx, hippocampus: HC.

To evaluate the relative distribution of aggregated Aβ in the hippocampus and neocortex of 1, 4, 6, and 12-month-old APP_SL_ mice, analyses were performed using the Amorfix A^4^ assay. The results showed a significant increase in levels of aggregated Aβ with age. The finding that graphs of the hippocampus and neocortex are nearly superimposable (Figure 2A) suggests a uniform distribution of aggregated Aβ in these two brain regions. The brain fibrillar Aβ content was investigated by the quantification of LOC immunoreactivity in the hippocampus and neocortex of 6, 9, and 12-month-old transgenic APP_SL_ mice. LOC is specific to a fibrillar amyloid structure and binds to mature and immature amyloid depositions. LOC labeling is more sensitive at measuring total plaque burden compared to classical plaque markers (6E10, 4G8, Congo red, and so on), thus a 2.5% IR area was detected at the young age of 6 months. Remarkably, our data also revealed high absolute levels of fibrillar Aβ and a significant and profound progression between 6 and 12-month-old APP_SL_ animals in both brain regions, and in the cortex also between 6 and 9 months (Figure 2B). No significant difference was measured between the hippocampus and neocortex. Representative images of LOC IR amyloid load and its spread over age are shown in Figure 2C. The absent LOC labeling in non-transgenic littermates is shown in Additional file 5.

### Oxidative stress markers increase over age in APP_SL_ mice

**Figure 3:**
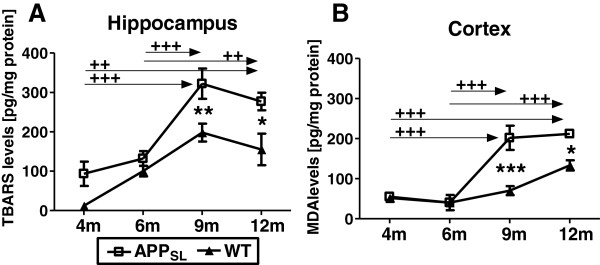
**Progression of oxidative stress in the hippocampus and neocortex of APP**_**SL **_**transgenic mice over age.** Lipid peroxidation is shown as TBARS levels per mg protein in the hippocampus **(A)** and neocortex **(B)** of 4, 6, 9, and 12-month-old APP_SL_ mice. n = 18/group. All data were analyzed by two way ANOVA followed by Bonferroni’s *post hoc* test. * significances between genotypes. **+** significances between age groups of APP_SL_ transgenic mice. *P <0.05; **P <0.01; ***P <0.001. Abbreviations: TBARS: thiobarbituric acid reactive substances, analysis of variance: ANOVA, Amyloid Precursor protein carrying the Swedish London mutation: APP_SL_, probability: P.

Oxidative stress measured by the lipid peroxidation indicator TBARS (TBARS assay) at the age of 4, 6, 9, and 12 months is shown for the hippocampus (Figure 3A) and the neocortex (Figure 3B) of APP_SL_ transgenic and non-transgenic littermates. TBARS levels increased moderately from 4 to 12 months in non-transgenic littermates. The relative increase was more pronounced in the hippocampal compared to cortical specimens. The increase in the neocortex between 4 and 12-month-old non-transgenic mice was significant. In APP_SL_ transgenic animals however, a profound increase in oxidative stress was observed between 6 and 9 months with essentially no further increase up to 12 months. This led to genotype-dependent significantly elevated TBARS levels in APP_SL_ transgenic animals at 9 and 12-months-old.

### Micro- and astrogliosis profoundly increase over age in APP_SL_ mice

**Figure 4:**
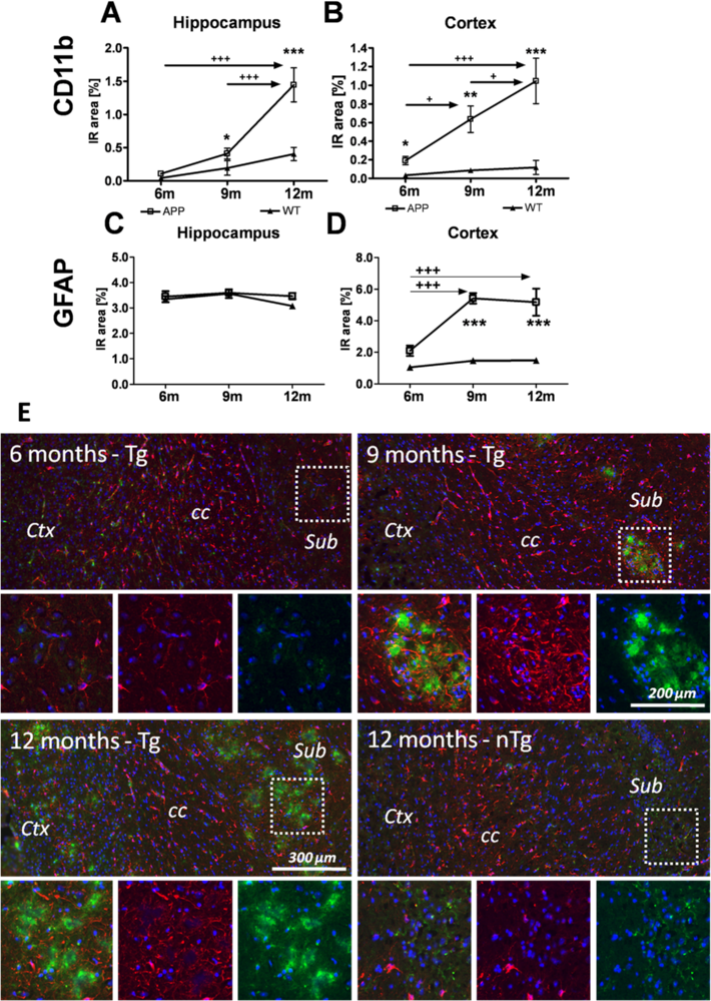
**Progression of microglia activation and reactive astrogliosis in different brain areas of APP**_**SL **_**transgenic mice over age.** Activated microglia (CD11b staining; **A**, **B**) and reactive astrocytes (GFAP staining; **C**, **D**) in the hippocampus **(A, C)** and neocortex **(B, D)** of 6, 9, and 12-month-old APP_SL_ mice are shown as the percentage of immunoreactive area (IR). n = 6/group. **E**: Representative images of CD11b (green) and GFAP (red) immunofluorescent double-labeling counterstained with DAPI in the subiculum of 6, 9, and 12-month-old APP_SL_ mice and a 12-month-old non-transgenic littermate. Note the absence of gliosis in the non-transgenic littermate, while micro- and astroglia typically progressively and concomitantly circumvent amyloid plaques in APP_SL_ mice at higher frequency with the increase of local plaque load. All data were analyzed by two way ANOVA followed by Bonferroni’s *post hoc* test. * significances between genotypes. **+** significances between age groups of APP_SL_ transgenic mice. *P <0.05; **P <0.01; ***P <0.001. Scale bar: 100 μm. Abbreviations: neocortex: Ctx, corpus callosum: cc, subiculum: Sub., glial fibrillary acidic protein: GFAP, DAPI: 4′,6-Diamidin-2-phenylindol, analysis of variance: ANOVA, Amyloid Precursor protein carrying the Swedish London mutation: APP_SL_, probability: P.

Histological quantification of CD11b staining in the hippocampus and neocortex of 6, 9, and 12-month-old APP_SL_ mice revealed significant microglia activation at all age groups compared to non-transgenic littermates (Figure 4A, B). Most notably, the increase is linearly progressive over age (Figure 4A, B). Cortical astrogliosis was genotype-dependently increased at 9 and 12 months, but not at 6 months. Astrogliosis levels increased significantly in APP_SL_ mice between 6 and 9 months and reached a plateau between 9 and 12 months (Figure 4D). In the hippocampus the amount of GFAP immunoreactivity detected in both APP_SL_ transgenic and non-transgenic littermates were similar and unchanged over age (Figure 4C). Representative images of CD11b and GFAP double-labeling of 6, 9, and 12-month-old APP_SL_ mice showed the age-related pronounced increase in neuroinflammation in the neocortex (Figure 4E).

### Correlations between Aβ levels, gliosis and oxidative stress

**Figure 5:**
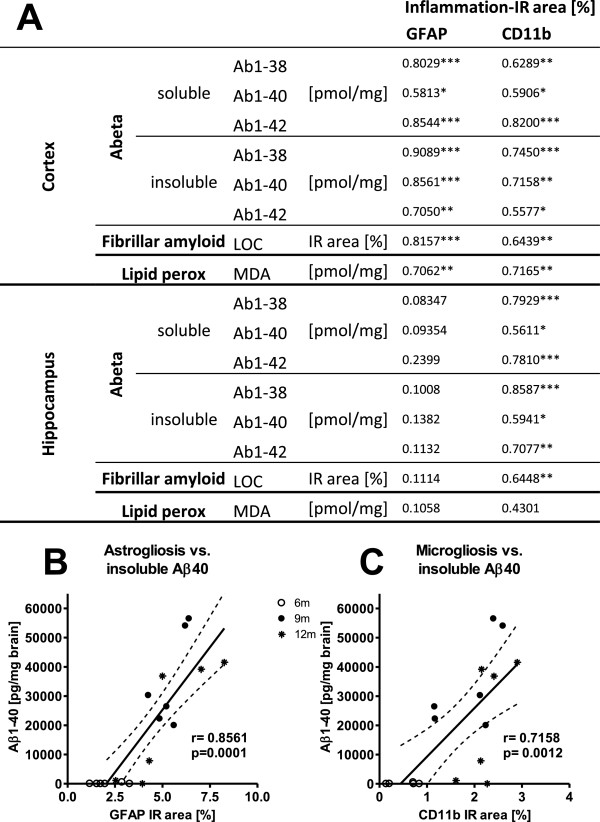
**Correlation analyses of neuropathological features of APP**_**SL **_**mice of all age groups. (A)** Correlation between neuroinflammation markers GFAP or CD11b and soluble or insoluble Aβ levels, fibrillar Aβ (LOC) or lipid peroxidation in the neocortex or hippocampus of APP_SL_ mice over age. Pearson’s correlation coefficient (r) and *P* value (*P <0.05; **P <0.01; ***P <0.001) are shown. Representative graphs of correlations between GFAP **(B)** or CD11b **(C)** and Aβ40 in the cortex over age are given. Abbreviations: glial fibrillary acidic protein: GFAP, amyloid beta: Aβ, analysis of variance: ANOVA, Amyloid Precursor protein carrying the Swedish London mutation: APP_SL_, probability: P.

Linear regression analyses (Pearson’s correlation) revealed a large number of significant variable dependencies. Analyses showed strong correlations in the neocortex between reactive microglia (CD11b values) or activated astrocytes (GFAP levels) and levels of Aβ (Figure 5A). For example the correlations between astrocytosis or microgliosis and soluble Aβ42 (r = 0.85, r = 0.82, respectively), insoluble Aβ38 (r = 0.9, r = 0.75, respectively) and Aβ40 (r = 0.86 (Figure 5B), r = 0.72 (Figure 5C), respectively) were highly significant. Interestingly, cortical microgliosis and astrocytosis also correlated significantly with both fibrillar Aβ levels and lipid peroxidation levels. In contrast, although hippocampal correlation coefficients were comparably high for activated microglia compared to levels of Aβ and fibrillar Aβ, no significant correlations were found between hippocampal microgliosis and lipid peroxidation, or between hippocampal astrogliosis and any markers.

### Progression of pathological hallmarks of APP_SL_ mice

**Figure 6:**
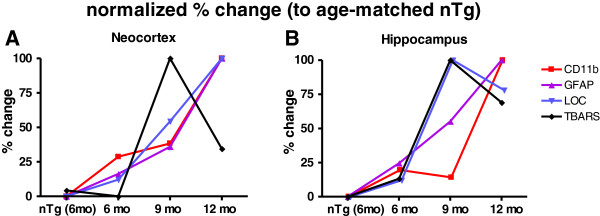
**Progression of pathological hallmarks in APP**_**SL **_**mice over age.** Graphs represent percent change of markers normalized to the values of age-matched non-transgenic littermates. Six-month-old non-transgenic littermates serve as baseline to show the change at the onset age. The maximum change during the investigated age span is set to 100%. Thus data display the dynamics of amyloid fiber load (LOC), activated microgliosis (CD11b), astrogliosis (GFAP) and lipid peroxidation (TBARS) for the neocortex **(A)** and the hippocampus **(B)**. Abbreviations: glial fibrillary acidic protein: GFAP, thiobarbituric acid reactive substances: TBARS, amyloid beta: Aβ, Amyloid Precursor protein carrying the Swedish London mutation: APP_SL_.

Data generated in this study were expressed as relative percent change compared to age-matched non-transgenic littermates, whereas the six-month-old non-transgenic group was plotted as a reference for the changes observed for the first correlatively investigated age. Figure 6 thus allows a comparison of events in a timely manner, and of the degree of change within each marker. At first sight the dynamics are notably different for the neocortex and the hippocampus.

In the neocortex, microglial activation increase to 25%, while astrogliosis and amyloid fiber load rise to approximately 12% at the age of 6 months. Increased lipid peroxidation cannot be observed at this age, thus it lags behind early amyloid deposition and inflammatory events. However, increasing amyloid levels and neuroinflammation towards 9 months of age are paralleled by an enormous increase of TBARS levels, while microglial activation does not follow with a strong change (Figure 6A).

In the hippocampus the profile is similar in regards to the start of microglial activation and amyloid load in the neocortex. However, the TBARS levels concomitantly increase together with LOC immunogenicity, without a time lag and strikingly parallel in the extent. Just as in the neocortex, lipid peroxidation is most prominent between 6 and 9 months of age, while again the microglial activation lags behind in the phase of increasing TBARS levels (Figure 6B). The concomitance of hippocampal TBARS levels with amyloidosis can also be observed in the correlation analyses. Whereas cortical TBARS levels are never correlated with any LOC immunogenicity, hippocampal TBARS levels significantly correlate with cortical levels (r = 0.69, *P* = 0.0039), as well as the hippocampal LOC load (r = 0.74, *P* = 0.0013).

## Discussion

The present study illustrates for the first time the progressive increase in AD-specific biochemical hallmarks in APP_SL_ mice, including profoundly increased soluble and insoluble Aβ40 and Aβ42 levels, aggregated Aβ, fibrillar Aβ, lipid peroxidation, and astro- as well as microgliosis in the hippocampus and neocortex up to an age of 12 months. Correlation analyses revealed a highly significant correlation between the severity of neuroinflammation and Aβ, fibrillar Aβ and lipid peroxidation levels.

Several studies in the past have demonstrated that Aβ40 and Aβ42 levels are elevated in the cortex of AD patients. This increase is even detectable in questionable dementia cases and correlates with the subsequently observed progression of cognitive decline [25]. As such, increasing Aβ40 and Aβ42 peptide concentrations in the brain seem to be useful indicators of developing dementia. When analyzing the different effects of soluble and insoluble Aβ peptides in the brains of AD patients, McLean *et al. *[26] identified soluble Aβ species as indicators for the severity of the disease. Similar results were obtained by Gong *et al. *[27] who unraveled the importance of small, soluble Aβ oligomers for AD pathology, suggesting that they might be appropriate targets for therapeutic AD drugs [27]. In fact, vaccination and immunization studies designed to reduce the Aβ peptide burden have led to learning and memory improvements in transgenic AD mice [28-30]. In contrast, the results from McLean *et al.* implied that amyloid plaque load serves only as a general marker of AD even under conditions when its total concentration is very high [26]. However, post mortem amyloid plaque analysis in humans is widely performed semi-quantitatively, which has a high variance between sampling, protocols and raters performing the assessments. These variable findings add to the methodological complexity of AD diagnosis and correlation analyses.

From the correlation data presented in this study we demonstrate that most measured variables increase in parallel which makes it impossible to incriminate one variable as the source, at least for the APP_SL_ mouse model. All analyzed variables, (aggregated Aβ, fibrillar Aβ, soluble and insoluble Aβ species) significantly increased over age and correlated with neuroinflammation markers. This implies that all these variables simultaneously contribute to AD disease progression. Havas *et al. *[21] previously analyzed the amyloid plaque load and number of plaques in APP mice with Swedish and London mutations, and observed the same progression for classical plaque markers (6E10 and Thioflavin S). Thus Aβ concentration, fibrillar Aβ and plaque load of APP_SL_ mice correspond to each other, as well as to cognitive decline, from 6 months of age onwards [21].

To date, the significance of plaque load for AD is still under debate. While several groups found the pattern of amyloid plaques to be of limited relevance for the neuropathological staging of AD [31-33], results from other laboratories demonstrated a strong correlation between counts of senile plaques in the hippocampus and cortex, and memory function such as the Blessed test for Information, Concentration, and Memory (BICM) in humans [34]. Recent findings describing Aβ deposition as a very slow process in humans, mostly occurring within the preclinical stage, and reaching a plateau as AD progresses, contradict a good correlation with the progressive memory decline in AD [35-37]. In contrast to this slow disease progression over years in humans [38], *in vivo* imaging of APP mice (Tg2576) has identified plaque formation and maturation as a fast process, occurring within weeks [38].

Microglial activation parallels Aβ plaque burden in AD patients and is thus a characteristic feature of AD progression [39,40]. This is consistent with the data presented here, where Aβ and Aβ fibril load strongly correlate with microgliosis. The modifiable nature of reactive microgliosis in these mice was previously shown by Imbimbo *et al.*[41] who treated the APP_SL_ mice with a γ-secretase modulator or ibuprofen as a positive control, resulting in a significant reduction of microglial inflammation [41]. Since corresponding correlation analyses were not performed, memory improvement cannot be associated with the decreased plaque load or neuroinflammation with certainty.

Beyond microgliosis, reactive astrocytes are the second major component of neuroinflammation in AD. A previous report demonstrated that affected patients present with a significant increase in astrocytosis in their hippocampal [42] and cortical areas [43] compared to non-demented patients. In our APP_SL_ mouse model we observed a profound increase only in neocortical but not in hippocampal reactive astrocyte levels up to an age of 12 months. This suggests that our mouse model, at first glance, does not exactly mimic the human phenotype. However, in both humans and APP_SL_ mice, Aβ plaques start to accumulate in the neocortex and only afterwards spread into the hippocampus. As such, it cannot be precluded that astrogliosis will be measurable in APP_SL_ mice at time points later than those evaluated in the current study.

Previous preclinical data demonstrated a successful pharmacological intervention which reduced astrogliosis in APP_SL_ mice [44]. Treatment of these animals with a cholesterol acyltransferase inhibitor caused, in addition to a reduction of amyloid plaque load and insoluble Aβ40 and Aβ42, a significant decrease in astrogliosis [44], suggesting that neuroinflammation in APP_SL_ mice is a susceptible target for AD treatment.

The importance of neuroinflammation for the development of AD pathology is still under debate since neuroinflammatory events are shown to promote Aβ clearance, whereas chronic neuroinflammatory events seem to promote Aβ accumulation [6-11]. Since the data presented here point towards an Aβ expression followed by neuroinflammatory events, microglia activation might indeed elevate Aβ clearance. If such an effect exists, it is not measurable in our Aβ quantification, but might be hidden due to extensive amyloid depositions. Other characterizations of AD mouse models are published that show pro-inflammatory markers, as well as microgliosis, that seem to be active even before amyloid pathology [45-48]. However, investigations such as Heneka’s *et al. *[46] lack markers for early immature amyloid deposition (such as LOC immunoreactive fibers) and focused on Aβ1-42 and Thioflavin S [46], which can typically be found in the core of mature plaque depositions at late AD stages. Others, such as the characterization by Abbas *et al. *[47] or Janelsins *et al. *[48] similarly show neuroinflammation markers to be increased in parallel or after Aβ expression. Although there may be some exceptions, for example Janelsins’ report of early inflammatory changes preceding amyloid pathology in the 3 × Tg-AD mouse model [48], the majority of studies demonstrate parallels in the timing of pro-inflammatory events and amyloid deposition [49]. In any case and independent of probable differences between different animal models, the start of amyloid deposition and gliosis is similar to humans, closely correlated and the connection could be instant and bidirectional [39,40,50,51].

The current understanding implies that oxidative stress is a hallmark of AD and able to activate inflammatory processes [12]. Oxidative damage is increased in patients with mild cognitive impairment (MCI) and early AD, highlighted by increased protein carbonyls, MDA [52], TBARS [53], and prolidase. Of particular interest is the finding that in AD patients an increased total oxidant status correlates negatively with results of the Mini mental state examination (MMSE) [54]. Accordingly, two additional publications on oxidative stress in MCI and AD patients define oxidative damage as the earliest events in AD development [55,56]. These data are further supported by results of Nunomura *et al. *[57] who analyzed the relationship between two oxidative damage markers and histological and clinical variables in AD individuals. Their findings clearly identified the most severe oxidative damage in early disease stages while decreasing with increasing Aβ accumulation [57].

Our evaluation of lipid peroxidation as a marker for oxidative stress in the neocortex of APP_SL_ mice seemingly contradicts hitherto findings in humans. Our data revealed a pronounced increase in lipid peroxidation [57], but lagging behind the significant amyloid deposition and concomitant astro- and microgliosis in the cerebral cortex. In the hippocampus, on the other hand, TBARS correlatively increased with LOC immunogenicity. These findings suggest that regional differences may exist which effect the biochemical reactions influencing lipid peroxidation, whereby the hippocampus plays a more prominent role.

Tissue storage under nitrogen gas would protect samples from subsequent oxidative events but was not used in this study. Thus, tissue storage might be a slight caveat since artificial changes in oxidative damage from tissue preparation might have occurred. However, since tissues of all animals of this study were sampled and stored in the same way, the potential change in oxidative damage is relative.

A common and interesting finding in the hippocampus and neocortex was the decreased microglia activation in the phase of strong increase of lipid peroxidation between 6 and 9 months of age. Notably this relation could shed light on a possible mechanism on how antioxidants such as resveratrol [58] could, for example, act by increasing microglial activation and enhanced microglia clearance of amyloid deposits by altering oxidative stress. Although, Capiralla *et al. *[59] as well as Solberg *et al. *[60] recently showed rather lower microglial activity which was parallel to decreased amyloid load [59,60], Solberg further claimed low potential of the antioxidative effect of resveratrol. The authors’ conclusion of microglial deactivation might have been carried too far, since both studies were evaluated after long treatment periods and not monitored during probably critical phases. Furthermore, the disappearance of microglia around amyloid plaques as measured by Capiralla *et al. *[59] might rather argue for activation towards a macrophagic and mobile M2 type, while the usual stickiness of microglia to amyloid plaques could rather reflect the problematical chronic inflammatory response and reduced amyloid clearance ability. Microglia activity might thus be down regulated in the study by Capiralla *et al.* simply due to the fact that TBARS is increased, which would support Solberg *et al.*’s finding of low antioxidant potential [59,60].

However, APP_SL_ mice first start to present significant cognitive deficits at the age of 6 months [61]. At this age, animals already have highly increased Aβ levels, neuroinflammation, and the appearance of lipid peroxidation differs between brain regions. Therefore, it can be concluded that lipid peroxidation in APP_SL_ mouse brains is not necessarily a prerequisite for the development of cognitive deficits, but fits concomitantly to hippocampal amyloid fiber deposition and hippocampal dependent learning tasks. As a challenging question for the future, it might be a rather consecutive event in the pathological cascade in neocortical regions. A parallel of hippocampal oxidative stress and symptomatic memory loss might even explain why symptomatically normal subjects can live with cortical amyloid load, while increased hippocampal oxidative stress could induce a seesaw of pathological events leading to the severe memory deficits observed.

## Conclusions

Inflammatory changes in the neocortex of APP_SL_ mice strongly correlate with levels of Aβ peptides, fibrillar Aβ, LOC immunoreactive Aβ, and lipid peroxidation, reflecting a strong interrelationship between all variables. Since APP_SL_ mice overexpress mutated APP, it is assumed that Aβ overexpression is the initiating event in the pathological cascade of AD specific hallmarks. Together with previously published data on progressive cognitive decline [21,22,61], the current study shows that APP_SL_ mice closely mimic the behavioral and neuropathological profile of AD patients, and might help to further elucidate the complex association of pathological factors in the disease.

## Abbreviations

Aβ: Amyloid beta; AD: Alzheimer’s disease; ANOVA: analysis of variance; APP: amyloid precursor protein; APPSL: amyloid precursor protein carrying the Swedish and London mutation; A4 assay: Amorfix Aggregated Aβ Assay; BHT: butylhydroxytoluene; BICM: Blessed Test for Information, Concentration, and Memory; cc: corpus callosum; cx: neocortex; DAPI: 4′,6-Diamidin-2-phenylindol; DEA: diethyl amine; FA: formic acid; GFAP: glial fibrillary acidic protein; HC: hippocampus; IR: immunoreactive; MCI: mild cognitive impairment; MDA: Malondialdehyde; MMSE: mini mental state examination; MSD: Meso Scale Discovery; p: probability; QPS: quest pharmaceutical services; SDS: sodium dodecyl sulfate; SEM: standard error of the mean; S/N: signal to noise; Sub: subiculum; TBARS: thiobarbituric acid reactive substances; THB: tissue homogenization buffer.

## Competing interests

SF, DH, CS, MW and BHP are employees of QPS-Austria GmbH. MU is an employee of Amorfix Life Sciences Ltd. The authors declare that they have no other competing interests.

## Authors’ contributions

TL designed, performed and analyzed measurements of Aβ levels, GFAP and CD11b stainings, TBARS assays and correlation analyses, prepared figures and edited the manuscript. SF prepared figures and wrote the manuscript. DH performed image analyses, correlation analyses and edited the manuscript. CS designed, performed and analyzed the A^4^ assay and edited the manuscript. MU developed the A^4^ assay and edited the manuscript. MW conceived of the study and participated in the design and interpretation of experiments. ES designed and interpreted experiments and edited the manuscript. BHP designed and interpreted experiments and edited the manuscript. All authors read and approved the final manuscript.

## Supplementary Material

Additional file 1 **Mediolateral sequence of sagittal sectioning levels.** Uniform, systematic random sets of ten sections per level covering the neocortex and hippocampal formation were collected from 12 mediolateral levels. Drawings taken from ‘The Mouse Brain in Stereotactic Coordinates’ by Paxinos and Franklin (2001, 2nd Edition). The sectioning starts with a random section at approximately 0.24 lateral from midline and extends uniformly and systematically throughout the whole hemisphere, always retaining 10 and discarding 20 sections per level. Levels 2, 4, 6, 8 and 11 were labeled [62]. Click here for file

Additional file 2 **Manual delineation of measured brain areas.** Anatomical figure taken from Paxinos & Franklin ‘The Mouse Brain Atlas’ showing the delineations of the regions defined as ‘cortex’ and ‘hippocampus’. Note that the boundaries for the hippocampus include the subiculum but exclude the white matter (fimbria) and that the cortex was defined as the neocortex excluding the accessory olfactory cortices, tubercle and amygdala and including the cingulate [62]. Click here for file

Additional file 3 **Brain neocortical and hippocampal region size.** Graphs show that there are no significant differences detectable between measured region sizes, thus the model does not suffer from atrophy within the scope of the used n and natural variance in neither the neocortex nor the hippocampus during the investigated range of age. It furthermore excludes relevant bias in sample processing. Click here for file

Additional file 4 **Aβ concentrations in cortical brain homogenates of APP**_**SL**_** transgenic mice over age.** Aβ38 (A, D); Aβ40 (B, E), and Aβ42 (C, F) concentrations are shown in pg/mg cortical homogenates for soluble (DEA; A-C) and insoluble (FA; D-F) fractions of 6, 9, and 12 month old APP_SL_ mice. N = 10 per group. All data were analyzed by two way ANOVA followed by Bonferroni’s *post hoc* test. * significances between genotypes. **+** significances between age groups of APP_SL_ transgenic mice. *P <0.05; **P <0.01; ***P <0.001. ^###^ significances between six-month-old APP_SL_ and non-transgenic littermates as analyzed by t-test (*P* <0.001). Analysis of the FA fraction of nine month old APP_SL_ mice for Aβ42 was not possible due to technical problems. Click here for file

Additional file 5 **LOC in non-transgenic APP**_**SL**_** littermates.** Representative images of an nTg littermate control animal at 12 months of age. Inlets show a piece of the neocortex (Cx) and the hippocampal subiculum (HC). Note that nTg do not show any kind of LOC labelling. Click here for file
