# Supplementary material for: Neuroinflammation and related neuropathologies in APPSL mice: further value of this in vivo model of Alzheimer’s disease
Source: J Neuroinflammation. 2014 May 1;11:84. doi: 10.1186/1742-2094-11-84 (PMC4108132; doi:10.1186/1742-2094-11-84)
Supplement: Additional file 1 — Mediolateral sequence of sagittal sectioning levels. Uniform, systematic random sets of ten sections per level covering the neocortex and hippocampal formation were collected from 12 mediolateral levels. Drawings taken from ‘The Mouse Brain in Stereotactic Coordinates’ by Paxinos and Franklin (2001, 2nd Edition). The sectioning starts with a random section at approximately 0.24 lateral from midline and extends uniformly and systematically throughout the whole hemisphere, always retaining 10 and discarding 20 sections per level. Levels 2, 4, 6, 8 and 11 were labeled [62]. [file 1742-2094-11-84-S1.pdf]

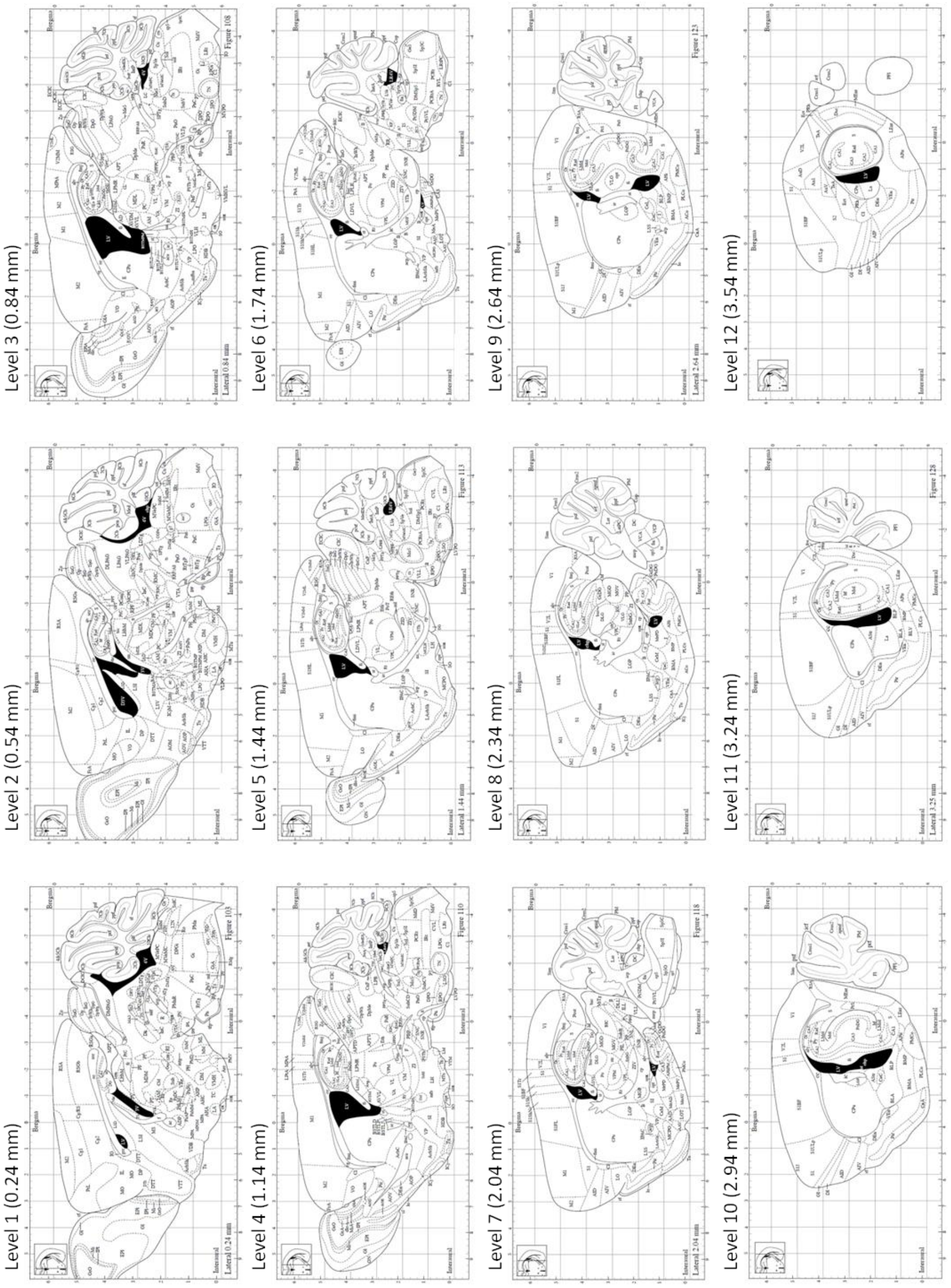

Add. File 2: Mediolateral sequence of sagittal sectioning levels. Uniform, systematic random sets of ten sections per level covering the neocortex and hippocampal formation were collected from 12 mediolateral levels. Drawings taken from “The Mouse Brain in Stereotactic Coordinates” by Paxinos and Franklin (2001, 2nd Edition).

The sectioning starts with a random section at approximately 0.24 lateral from midline and extends uniformly and systematically throughout the whole hemisphere, always retaining 10 and discarding 20 sections per level.

Levels 2, 4, 6, 8 and 11 were labeled.
