# Supplementary material for: Neuroinflammation and related neuropathologies in APPSL mice: further value of this in vivo model of Alzheimer’s disease
Source: J Neuroinflammation. 2014 May 1;11:84. doi: 10.1186/1742-2094-11-84 (PMC4108132; doi:10.1186/1742-2094-11-84)
Supplement: Additional file 2 — Manual delineation of measured brain areas. Anatomical figure taken from Paxinos & Franklin ‘The Mouse Brain Atlas’ showing the delineations of the regions defined as ‘cortex’ and ‘hippocampus’. Note that the boundaries for the hippocampus include the subiculum but exclude the white matter (fimbria) and that the cortex was defined as the neocortex excluding the accessory olfactory cortices, tubercle and amygdala and including the cingulate [62]. [file 1742-2094-11-84-S2.pdf]

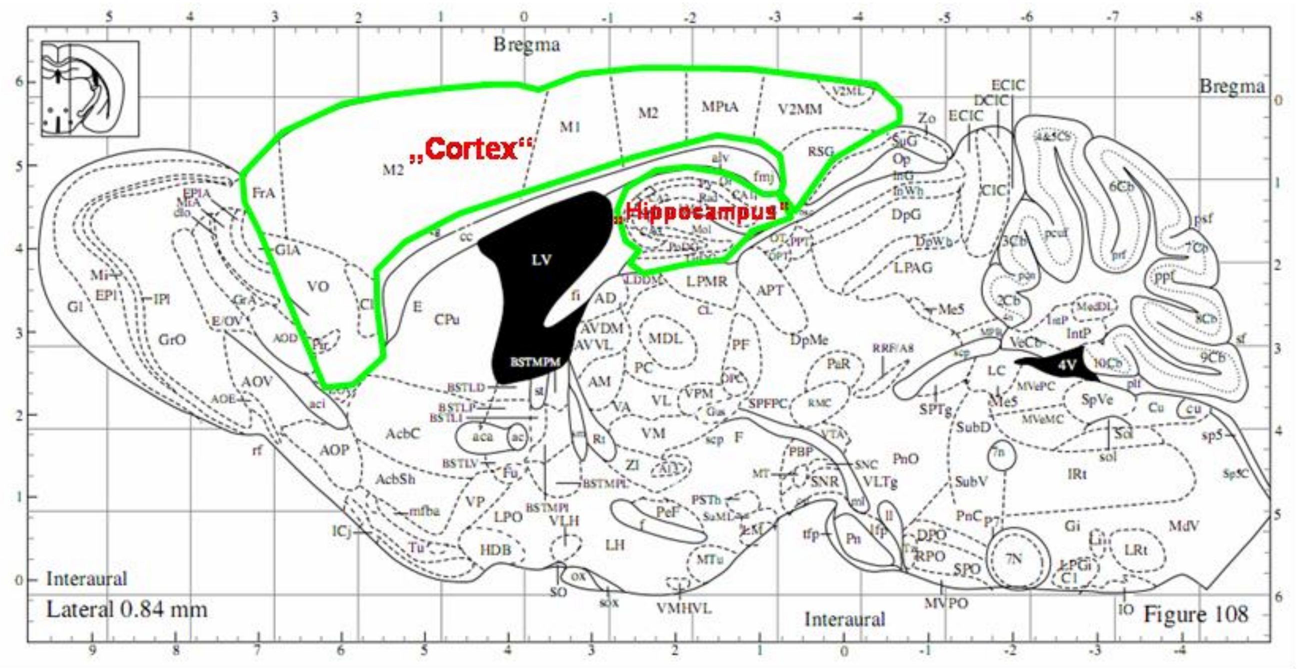

Add. File 3: Manual delineation of measured brain areas. Anatomical figure taken from Paxinos & Franklin "The Mouse Brain Atlas" showing the delineations of the regions defined as "cortex" and "hippocampus". Note that the boundaries for the hippocampus include the subiculum but exclude the white matter (fimbria) and that the cortex was defined as the neocortex excluding the accessory olfactory cortices, tubercle and amygdala and including the cingulate.
