# Supplementary material for: Neuroinflammation and related neuropathologies in APPSL mice: further value of this in vivo model of Alzheimer’s disease
Source: J Neuroinflammation. 2014 May 1;11:84. doi: 10.1186/1742-2094-11-84 (PMC4108132; doi:10.1186/1742-2094-11-84)
Supplement: Additional file 3 — Brain neocortical and hippocampal region size. Graphs show that there are no significant differences detectable between measured region sizes, thus the model does not suffer from atrophy within the scope of the used n and natural variance in neither the neocortex nor the hippocampus during the investigated range of age. It furthermore excludes relevant bias in sample processing. [file 1742-2094-11-84-S3.pdf]

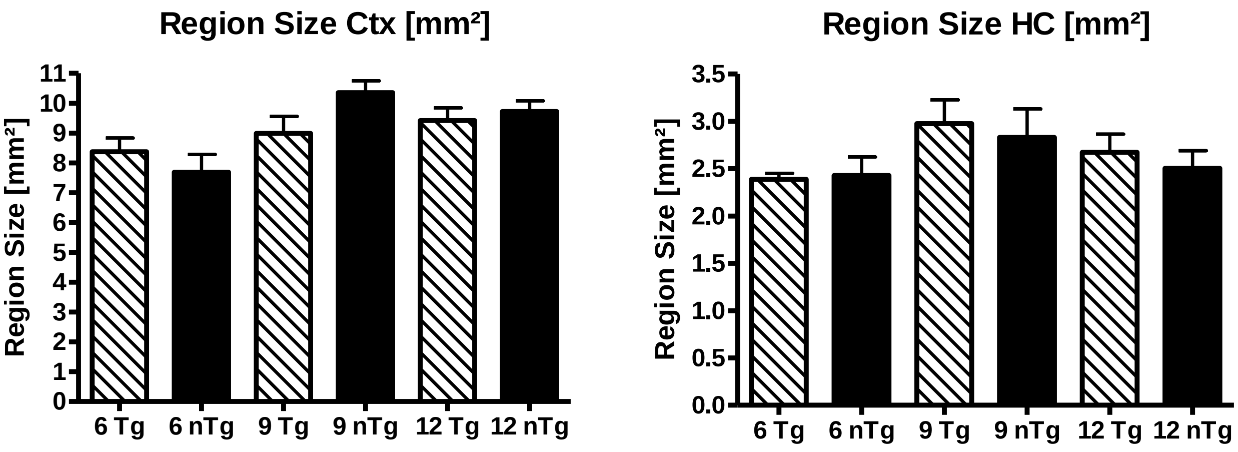

Add. File 4: Brain neocortical and hippocampal region size. Graphs show that there are no significant differences detectable between measured region sizes, thus the model does not suffer from atrophy within the scope of the used n and natural variance in neither the neocortex nor the hippocampus during the investigated range of age. It furthermore excludes relevant bias in sample processing.
