# Supplementary material for: Neuroinflammation and related neuropathologies in APPSL mice: further value of this in vivo model of Alzheimer’s disease
Source: J Neuroinflammation. 2014 May 1;11:84. doi: 10.1186/1742-2094-11-84 (PMC4108132; doi:10.1186/1742-2094-11-84)
Supplement: Additional file 4 — Aβ concentrations in cortical brain homogenates of APPSL transgenic mice over age. Aβ38 (A, D); Aβ40 (B, E), and Aβ42 (C, F) concentrations are shown in pg/mg cortical homogenates for soluble (DEA; A-C) and insoluble (FA; D-F) fractions of 6, 9, and 12 month old APPSL mice. N = 10 per group. All data were analyzed by two way ANOVA followed by Bonferroni’s post hoc test. * significances between genotypes. + significances between age groups of APPSL transgenic mice. *P <0.05; **P <0.01; ***P <0.001. ### significances between six-month-old APPSL and non-transgenic littermates as analyzed by t-test (P <0.001). Analysis of the FA fraction of nine month old APPSL mice for Aβ42 was not possible due to technical problems. [file 1742-2094-11-84-S4.pdf]

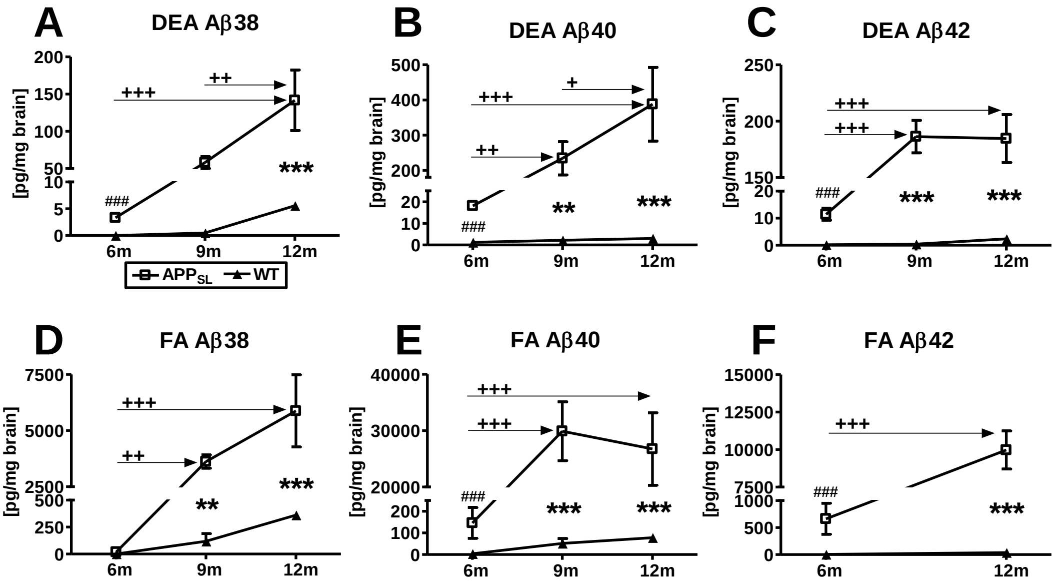

Add. File 1: Aβ concentrations in cortical brain homogenates of APP$_{SL}$ transgenic mice over age. Aβ38 (**A, D**); Aβ40 (**B, E**), and Aβ42 (**C, F**) concentrations are shown in pg/mg cortical homogenates for non-plaque (DEA; **A-C**) and plaque associated (FA; **D-F**) fractions of 6, 9 and 12 month old APP$_{SL}$ mice. N = 10 per group. All data were analyzed by two way ANOVA followed by Bonferroni's post hoc test. * significances between genotypes. **+** significances between age groups of APP$_{SL}$ transgenic mice. *p<0.05; **p<0.01; ***p<0.001. ### significances between 6 month old APP$_{SL}$ and non-transgenic littermates as analyzed by t-test (p<0.001). Analysis of the FA fraction of 9 month old APP$_{SL}$ mice for Aβ42 was not possible due to technical problems.
