# Supplementary material for: Neuroinflammation and related neuropathologies in APPSL mice: further value of this in vivo model of Alzheimer’s disease
Source: J Neuroinflammation. 2014 May 1;11:84. doi: 10.1186/1742-2094-11-84 (PMC4108132; doi:10.1186/1742-2094-11-84)
Supplement: Additional file 5 — LOC in non-transgenic APPSL littermates. Representative images of an nTg littermate control animal at 12 months of age. Inlets show a piece of the neocortex (Cx) and the hippocampal subiculum (HC). Note that nTg do not show any kind of LOC labelling. [file 1742-2094-11-84-S5.pdf]

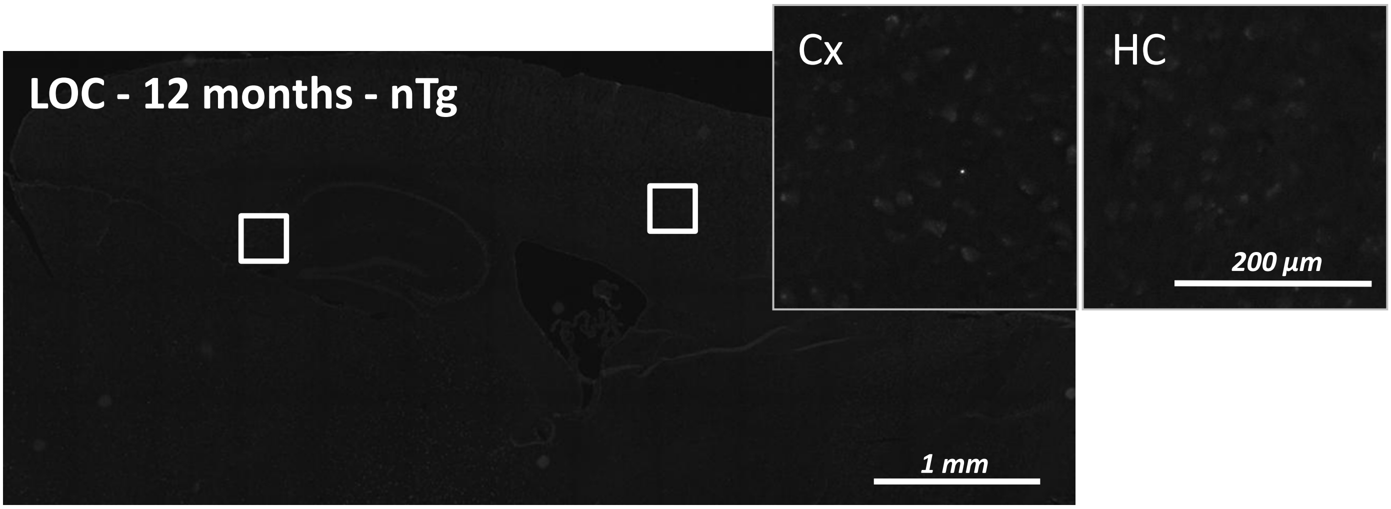

Add. File 5: LOC in non-transgenic APP_{SL} littermates. Representative images of an nTg littermate control animal at 12 months of age. Inlets show a piece of the neocortex (Cx) and the hippocampal subiculum (HC). Note that nTg do not show any kind of LOC labeling.
